# A method for addressing right upper lobe obstruction with right-sided double-lumen endobronchial tubes during surgery: a randomized controlled trial

**DOI:** 10.1186/s12871-018-0596-3

**Published:** 2018-09-18

**Authors:** Wei Yu, Zijian Wang, Dapeng Gao, Wei Zhang, Wen Jin, Xuesong Ma, Sihua Qi

**Affiliations:** Department of Anaesthesiology, Fourth Affiliated Hospital of Harbin Medical University, 37 Yiyuan Road, Harbin, 150001 Heilongjiang Province China

**Keywords:** airway management methods, one-lung ventilation, adverse effects, intubation

## Abstract

**Background:**

A right-sided double-lumen tube (R-DLT) tends to obstruct the right upper lobe intraoperatively due to anatomical distortion during surgery. If the R-DLT is poorly matched with the patient’s airway anatomy, it will not be possible to correctly replace the tube with a fiberoptic bronchoscope (FOB). In our study, we aimed to explore an efficient method for difficult repositioning caused by right upper lobe occlusion during surgery: repositioning the R-DLT from the right main bronchus into the left main bronchus. The current study was designed to assess the efficacy and safety of this method.

**Methods:**

Sixty adult patients scheduled to undergo left-sided thoracic surgery were randomly assigned to two groups. With the patient in the right lateral position during surgery, the R-DLT was pulled back to the trachea while being rotated 90° clockwise; it was then either rotated 90° clockwise for placement into the left main bronchus (Group L) or rotated 90° anticlockwise and returned to the right main bronchus (Group R) using FOB guidance. The primary outcomes included clinical performance, which was measured by intubation time, and the quality of lung collapse. A secondary outcome was safety, which was determined according to bronchial injury and vocal cord injury.

**Results:**

The median intubation time (IQR [range]) required for placement of a R-DLT into the left main bronchus was shorter than the time required for placement into the right main bronchus (15.0 s [IQR, 12.0 to 20.0 s]) vs 23.5 s [IQR, 14.5 to 65.8 s], P = 0.005). The groups showed comparable overall results for the quality of lung collapse during the total period of one-lung ventilation (P = 1.000). The numbers of patients with bronchial injuries or vocal cord injuries were also comparable between groups (Group R, 11/30 vs. Group L 8/30, P = 0.580 for bronchus injuries; Group R, 15/30 vs. Group L 13/30, P = 0.796 for vocal cord injuries).

**Conclusions:**

Repositioning a R-DLT from the right main bronchus into the left main bronchus had good clinical performance without causing additional injury. This may be an efficient method for the difficult repositioning of a R-DLT due to right upper lobe occlusion during surgery.

**Trial registration:**

Chinese Clinical Trial Registry, ChiCTR-IPR-15006933, registered on 15 August 2015.

**Electronic supplementary material:**

The online version of this article (10.1186/s12871-018-0596-3) contains supplementary material, which is available to authorized users.

## Background

In left-sided thoracic surgery, one-lung ventilation (OLV) can generally be achieved by placing a right- or left-sided double-lumen tube (R-DLT or L-DLT). A R-DLT has a smaller margin of safety for correct placement than a L-DLT [1]. The main problem with using a R-DLT is that it is prone to cause right upper lobe obstruction and collapse [2–4]. Nevertheless, many clinicians choose a R-DLT for left-sided thoracic surgery because the consensus is that the thoracic surgical procedure may change at any time, and for some procedures, using a R-DLT is absolutely indicated, such as in sleeve resection and left-sided pneumonectomy [5]. However, it has been found that during some operations, a R-DLT is not actually necessary.

A R-DLT has a tendency to become malpositioned intraoperatively [6] due to anatomical distortion that may occur during lateral positioning of the patient [7]. If a R-DLT is poorly matched with the anatomy of the patient’s airway, it will not be possible to replace the R-DLT correctly using a fiberoptic bronchoscope (FOB) even with repeated attempts, and multiple attempts to replace a R-DLT can lead to severe airway injury. Although these problems can be corrected by exchanging a R-DLT with a L-DLT or by using an endobronchial blocker, exchanging a DLT with the patient in the lateral position is not only difficult but also risks losing access to the airway.

The distance between the branching point of the left upper lobe orifice and the carina is approximately 4.5 cm in women and 5.0 cm in men [1, 8]. For most patients, the size of the left main bronchus is long enough to hold the tip of a R-DLT without occluding the left upper lobe orifice. Thus, transferring a R-DLT from the right main bronchus into the left main bronchus may be a feasible method for instances where R-DLT repositioning is difficult due to right upper lobe occlusion during surgery. Because the performance of this method is controversial, we performed a prospective, randomized controlled trial to test the efficacy and safety of moving a R-DLT from the right main bronchus into the left main bronchus with patients in the lateral position during surgery.

## Methods

### Patients and groups

This single-center, controlled, randomized, single-blinded study was approved by the Ethics Committee of The Fourth Affiliated Hospital of Harbin Medical University, Harbin, China (Chairperson Prof Changjiu Zhao) (No. YLXJS.2015.02) on 30 July 2015. The study protocol was registered with the Chinese Clinical Trial Registry (ChiCTR-IPR-15006933).

After providing written informed consent, adult patients with American Society of Anesthesiologists physical status classifications of 1 to 3 and aged 18–75 years were enrolled in our study. All of the patients underwent left-sided video-assisted thoracoscopic surgery (VATS) (e.g., lung biopsy, lobectomy, segmentectomy, pleural decortication) requiring OLV. We excluded patients for which we anticipated difficulties in tracheal intubation (e.g., a Mallampati score ≥ 3), a Cormack score of 3 or 4, the presence of a tracheostomy, pre-existing sore throat or hoarseness, or recent respiratory infection (< 1 month).

All of the recruited patients were allocated 1:1 into two groups using computer-generated randomization, and the group allocation number was hidden in opaque envelopes. To ensure blinding, three anesthesiologists were involved in the study. The first anesthesiologist performed anesthesia and the first bronchial intubation with the patient in the dorsal decubitus position. The second anesthesiologist opened the opaque envelope and performed the second tracheal intubation with the patient in the lateral decubitus position. The third anesthesiologist evaluated and recorded all of the parameters. Only the second anesthesiologist knew the group allocation of the study.

### Anesthetic management

The patients, who were not given any premedication, were transferred to the operating room. A catheter was placed in the radial artery for arterial blood gas analysis and continuous monitoring of blood pressure. All of the patients received a thoracic epidural catheter at the level of T_4–5_ or T_5–6_ for postoperative pain management.

After the induction of anesthesia with injections of 0.3 μg·kg^− 1^ sufentanil, 0.2 mg·kg^− 1^ cisatracurium, and 1.5–2.5 mg·kg^− 1^ propofol, all patients were preoxygenated with 100% oxygen for 3 min. A laryngeal mask airway (LMA) was inserted after lubricating the cuff with a water-based jelly. A bronchoscope was advanced down the LMA, and then, a pulmonologist observed the airway condition and measured the length of the left main bronchus with the bronchoscope. A video was also obtained for subsequent analysis and comparison [4].

After removal of the LMA, the Cormack-Lehane classification was used for quantification via direct laryngoscopy [9]. A disposable polyvinyl chloride R-DLT (Mallinckrodt Medical, Inc., Athlone, Ireland) without a carinal hook was used for tracheal intubation, and the size was selected according to patient gender, height and chest CT scan by a separate anesthetist. After the bronchial tip passed the vocal cords, the stylet was removed. The tube was rotated 90° clockwise and advanced until the estimated depth of insertion was reached. The accuracy of R-DLT placement was assessed with a FOB. The pressure of the R-DLT cuffs was measured with a noninvasive manometer (Endotest; Rüsch, Kernen, Germany) and maintained below 30 mmHg.

Anesthesia was maintained with sevoflurane (2–4% end-tidal concentration) and 0.25–1.0 μg·kg^− 1^·min^− 1^ remifentanil. In addition, 0.5–2 μg·kg^− 1^·min^− 1^ cisatracurium was continuously administered i.v. to maintain muscular blockade, and neuromuscular blockade was assessed by train-of-four monitoring. Volume-controlled mode was used with the following parameters: the tidal volume was 5–7 ml·kg^− 1^ (ideal body weight) during OLV and 8 ml·kg^− 1^ (ideal body weight) during two-lung ventilation, and respiration rates were regulated according to the end-tidal carbon dioxide tension within 35–45 mmHg. The inspired oxygen concentration was 100% before OLV initiation and was gradually reduced to maintain an oxygen saturation (SaO_2_) greater than 95%.

After performing two-lung ventilation for 15 min with the patient in the supine position, the following variables were recorded as the basal values: mean arterial pressure, heart rate, arterial blood oxygen tension, and pulmonary mechanics (peak pressure, plateau pressure, and dynamic compliance). After the bronchial cuff was deflated, the patient was move into the right lateral position, and the head was fixed in place. Then, the R-DLT was pulled back 6 cm to the trachea while being rotated 90° clockwise in the right lateral position (rotating the tip of the bronchial lumen towards the posterior side of the trachea). A FOB was advanced down the bronchial lumen. Then, the R-DLT was either rotated 90° clockwise while being guided via the FOB to the left main bronchus (left intubation group) or rotated 90° anticlockwise while being guided via the FOB back to the right main bronchus (right intubation group). The anesthesiologist was at the patient’s head, and the descriptions referring to clockwise/anticlockwise are relative to the orientation of the bronchoscopist. The placement of the R-DLT was adjusted with a FOB, and the resistance while placing the R-DLT was recorded. After completing the intubation procedure, we recommended leaving the R-DLT free to rotate to a lower rotation resistance. In our preliminary experiments, we found that the rotation resistance was high when the tube was rotated with the tip facing the anterior wall, which may more easily cause the DLT to twist in the airway or damage the airway during rotation. The reasons for the high rotation resistance that occurred might be best explained as follows. The R-DLT was initially rotated 90° clockwise after the bronchial tip passed the vocal cords. If the rotation direction was not consistent with the first rotation direction, it was more likely to lead to distortion of the R-DLT and increase rotation resistance. After the initiation of OLV, the pulmonary mechanics were recorded at 5 min, 15 min, and 30 min, and the arterial blood gas values were recorded before and 10 min after lung collapse.

After completing the surgery, the R-DLT was removed, and the laryngeal mask was inserted again. A bronchoscope was advanced down the LMA, and the pulmonologist re-assessed the airway condition with a bronchoscope to check for injuries caused by the R-DLT. Vocal cord injuries were recorded with video and assessed by an otolaryngologist. Airway injuries were defined according to methods described by Knoll [10–12]. For postoperative pain therapy, a mixture of 0.125% ropivacaine and 0.25 μg·ml^− 1^ sufentanil was given by epidural patient-controlled analgesia.

### Intubation variables and efficacy of the R-DLT

The following intubation-related variables were evaluated and recorded by the anesthesiologist: visualization of glottic exposure (Cormack-Lehane grade); time for the first tracheal intubation with the patient in the supine position, defined as the time from when the laryngoscope was placed into the mouth until the end-tidal carbon dioxide level was detected; time for the second tracheal intubation with the patient in the lateral position, which was the primary outcome of the study and was defined as the time from the beginning of tube withdrawal from the bronchial lumen to the trachea until successful intubation; and the number of intubation attempts. In addition, the ease of R-DLT placement (easy/difficult/very difficult) was also recorded for the second tracheal intubation.

According to the anatomy and pulmonary mechanics, the tube position was evaluated, and the observations were grouped into four categories: optimal, which consisted of a clear and unobstructed view of the main carina and subcarina; suboptimal, which consisted of unobstructed airways (without increased peak inspiratory pressure) and partly visible main carina or subcarina; misplaced, which consisted of acceptable mechanical ventilation and a nonvisible main carina or subcarina; and unsatisfactory, which consisted of inadequate complete mechanical ventilation and a nonvisible main carina or subcarina. The surgeon, who was blinded to the group allocation of the study, assessed the extent of lung collapse [4]: 1, excellent (complete collapse); 2, fair (some residual air); and 3, poor (no collapse or residual air interfering with surgical exposure).

### Assessment of postoperative hoarseness and sore throat

Hoarseness was defined as acoustic quality assessed according to the difference relative to the patient’s previous voice quality [12]. This variable was graded as mild (as deemed by the patient), moderate (as noticed by the observer), or severe (aphonia). Sore throat was defined as continuous pain in the throat [3, 13] and was rated as follows: mild (pain with swallowing), moderate (continuous pain and increased pain while swallowing), or severe (pain interfering with eating and requiring painkillers). The anesthesiologist asked each patient specific questions regarding his or her postoperative sore throat and hoarseness at 1 h after emergence from anesthesia and at 24 h, 48 h, and 72 h [12, 14].

### Statistical analysis

The sample size was calculated based on the intubation time. We defined a clinically important difference in intubation time as 10 s; therefore, using a standard deviation of 12 s based on a pilot study, an α = 0.05 and β = 0.2, 46 patients (23 patients in each group) were required. Considering potential dropouts, 30 patients were included in each group.

Continuous variables were tested for a normal distribution using the Kolmogorov–Smirnov test. Normally distributed continuous data, presented as the mean (standard deviation), were compared using independent Student’s *t*-tests. Non-normally distributed continuous data, presented as the median (interquartile range, IQR), were analyzed using the Mann–Whitney rank sum test. Numbers (%) for categorical variables were compared using Fisher’s exact test. The results were considered statistically significant when P was less than 0.05. Statistical analysis was performed using SPSS Statistics 18.0 (IBM Corp., Armonk, NY).

## Results

**Fig. 1:**
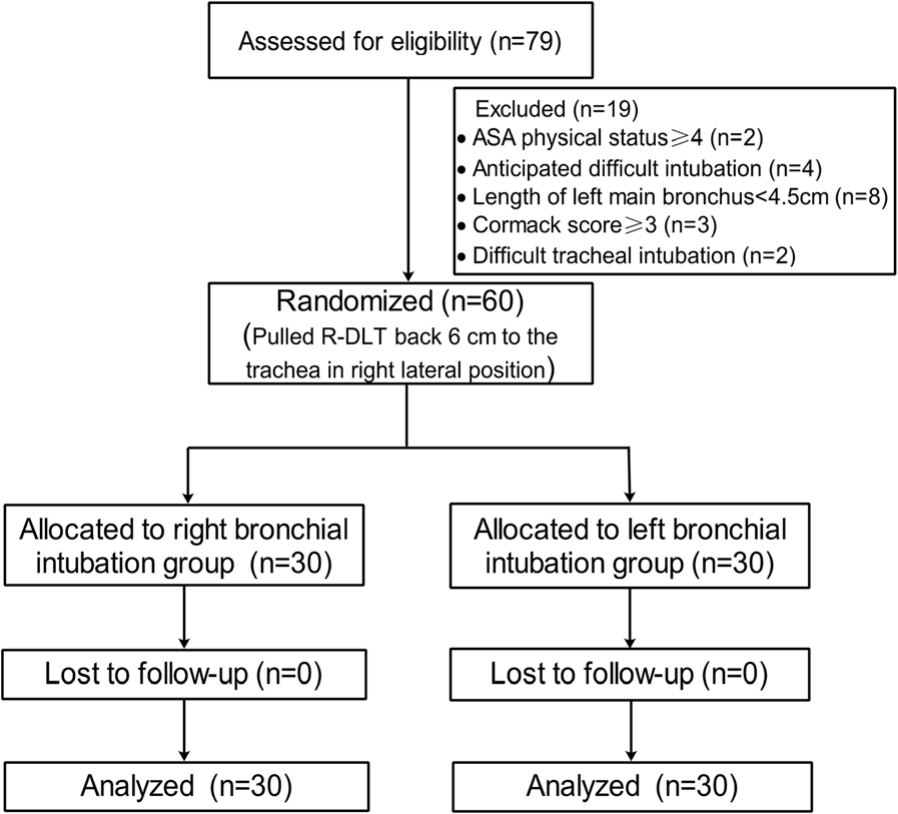
Experimental Flowchart. Study flow chart. ASA, American Society of Anesthesiologists physical status; R-DLT, right-sided double-lumen tube

During the period from August 2015 to June 2016, 79 patients were initially assessed for eligibility. Nineteen patients were excluded, and 60 patients were randomized (30 patients in each group) and completed the study (Fig. 1). Demographic and surgical data for the groups are summarized in Table 1.

**Table 1:** Patient characteristics and surgical data

	Group R (*n* = 30)	Group L (*n* = 30)
Age (years)	56.8 (9.8)	59.9 (8.8)
Male	19	21
Weight (kg)	65.5 (11.1)	64.6 (11.9)
Height (cm)	169.1 (5.9)	168.7 (8.1)
ASA physical status (I/II/III)	6/21/3	7/20/3
Smoking, Y/N	14/16	12/18
Cormack-Lehane view (1/2)	25/5	22/8
DLT size (FG), 35/37/39	12/16/2	14/13/3
Duration of surgery (min)	198.9 (54.2)	175.8 (37.0)

*ASA* American Society of Anesthesiologists physical status

Factors related to tracheal intubation did not differ significantly between the study groups for the initial tracheal intubation when in the dorsal decubitus position (Additional file 1: Table S1). For the second tracheal intubation in the lateral decubitus position, placement of the R-DLT into the left bronchus required a median duration of 15.0 s (IQR, 12.0 to 20.0 s), and placement of the R-DLT to the right bronchia required a median duration of 23.5 s (IQR, 14.5 to 65.8 s) (*P* = 0.005). Compared with right bronchial intubation, R-DLT placement into the left bronchus was rated as easier by the anesthesiologist with the patient in the lateral decubitus position (*P* < 0.001) (Table 2). Only 70% of right bronchial intubations and 26.7% of left bronchial intubations were initially placed in an optimal position. However, the total number of intubations in the proper position (optimal and suboptimal positions) without the need for repositioning were comparable in both groups (Group R, 26/30 vs. Group L, 27/30; *P* = 1.000) (Table 2). Additionally, the values for blood gas parameters, pulmonary mechanics, and quality of collapse were similar between the groups and are summarized in Table 3 and Additional files 2 and 3: Tables S2 and S3.

**Table 2:** Factors related to the second tracheal intubation in the lateral decubitus position

	Group R (*n* = 30)	Group L (*n* = 30)	*P* value
Time required for tracheal intubation and R-DLT positioning(s)	23.5 (14.5–65.8)	15.0 (12.0–20.0)	0.005^a^
Position after intubation (optimal/suboptimal/misplaced/ unsatisfactory)	21/5/4/0	8/19/2/1	> 0.001^b^
Number of tracheal intubation attempts (1/2/3)	25/4/1	27/2/1	0.832^b^
Ease of placing the R-DLT (easy/difficult/very difficult)	19/8/3	27/3/0	> 0.001^b^
Number of repositions (0/1/2/3)	22/6/1/1	24/3/1/2	0.794^b^
Patients^c^	26	27	1.000^b^

^a^Median (IQR) of the non-normal variables (Kolmogorov–Smirnov test, *P* < 0.05); significance tested by the Mann–Whitney rank sum test

^b^Numbers for the categorical variables; significance tested by Fisher’s exact test

^c^Number of patients with optimal or suboptimal positioning of the bronchial tubes

**Table 3:** Quality of collapse

	Group R (*n* = 30)	Group L (*n* = 30)	*P* value
Quality of collapse after 10 min, n			0.430^a^
Excellent	10	14	
Fair	19	16	
Poor	1	0	
Quality of collapse overall, n			1.000^a^
Excellent	25	26	
Fair	5	4	
Poor	0	0	

^a^Numbers for the categorical variables; significance tested by Fisher’s exact test

The overall incidence of bronchial injury was 32% (19 patients) for Group R and Group L combined. The intensity of bronchial injury did not differ significantly between the groups (Table 4) (*P* = 0.824). The number of patients with vocal cord injuries did not significantly differ between the two groups (*P* = 0.796). The overall incidence of vocal cord injury was 47% (28 patients), and the majority of the vocal cord injuries included redness (16 patients) and edema (9 patients) (Table 4).

**Table 4:** Incidence of bronchial injuries and vocal cord injuries

	Group R (*n* = 30)	Group L (*n* = 30)	*P* value
Bronchial injuries			0.824^a^
None	19	22	
Redness	8	6	
Edema	2	1	
Hematoma	1	1	
Patients^b^	11	8	0.580^a^
Vocal cord injuries			0.176^a^
None	15	17	
Redness	9	7	
Edema	5	4	
Hematoma	1	2	
Patients^b^	15	13	0.796^a^

^a^Numbers for the categorical variables; significance tested by Fisher’s exact test

^b^Number of patients with bronchial injuries or vocal cord injuries

The overall incidence of postoperative hoarseness was 27% (16 patients) at 1 h, 17% (10 patients) at 24 h, 13% (8 patients) at 48 h, and 5% (3 patients) at 72 h (Table 5), and these last 3 patients recovered from hoarseness on postoperative day 4. The presence of sore throat was similar between Group R and Group L: 11 patients vs. 14 patients experienced this outcome (*P* = 0.601) (Table 5). The overall incidences of sore throat were highest at 1 h and decreased thereafter. No patients suffered from sore throat on postoperative day 5.

**Table 5:** Incidence and intensity of postoperative hoarseness and sore throat

	Hoarseness			Sore throat		
	Group R (*n* = 30)	Group L (*n* = 30)	*P* Value	Group R (*n* = 30)	Group L (*n* = 30)	*P* value
At 1 h			0.481			0.491^a^
No complaints	23	21		21	18	
Mild	3	2		3	5	
Moderate	3	5		4	5	
Severe	1	2		2	2	
At 24 h			0.154			0.503^a^
No complaints	27	23		24	22	
Mild	2	3		3	3	
Moderate	1	4		2	3	
Severe	0	0		1	2	
At 48 h			0.445			0.666^a^
No complaints	27	25		28	27	
Mild	2	3		0	1	
Moderate	1	2		1	1	
Severe	0	0		1	1	
At 72 h			0.557			0.57^a^
No complaints	29	28		29	28	
Mild	1	2		0	1	
Moderate	0	0		1	1	
Severe	0	0		0	0	
Patients^b^	9	10	1.000	11	14	0.601^a^

^a^Numbers for the categorical variables; significance tested by Fisher’s exact test

^b^Numbers of patients with postoperative hoarseness and sore throat

## Discussion

The use of a R-DLT is related to a high risk of right upper lobe obstruction and collapse [2–4]. However, there are also some absolute indications for using a R-DLT, such as for treating left endobronchial tumors, disruption of the left mainstem bronchus, left lung transplantation, sleeve resection, left-sided pneumonectomy, and left bronchial compression of a thoracic aortic aneurysm. Due to the uncertainty in surgical procedures and patient conditions, a R-DLT is initially preferred for left-sided thoracic surgery in a number of clinical institutions. To the best of our knowledge, only a few case reports have described attempts at right bronchial intubation using a left-sided double-lumen tube [13, 15]. There are no previous prospective investigations that evaluated the efficiency and safety of repositioning a R-DLT from the right main bronchus into the left main bronchus with patients in the lateral position during surgery. Therefore, we designed a study to assess the efficiency and safety of this bronchial intubation technique.

The procedure for placement of a R-DLT in the left mainstem bronchus was rated as being easier and faster than that for placement in the right mainstem bronchus, mainly because R-DLT placement requires proper positioning of the R-DLT, with the ventilation slot opening directly into the bronchial orifice of the right upper lobe. Placement of R-DLTs is easy in most patients but can be troublesome in a subset of patients. After successful bronchial intubation when in the dorsal decubitus position, some patients are moved into the right lateral decubitus position, with R-DLT malpositioning detected via FOB, indicating a lack of alignment between the right upper lobe R-DLT ventilation slot and the origin of the right upper bronchus. It is sometimes not possible to replace a R-DLT correctly despite many attempts, and multiple attempts may cause serious airway damage. At the same time, subsequent operative procedures may not require a R-DLT. In these situations, the use of a R-DLT would still provide adequate surgical conditions if the R-DLT is repositioned into the left main bronchus.

Only 26.7% of left bronchial intubations were positioned optimally after the second tracheal intubation with the patient in the lateral decubitus position. The reasons for the high rate of malpositioning reported here might be best explained as follows. First, when the R-DLT was rotated 180° clockwise and guided to the left main bronchus via the FOB, the tube was prone to rotate slightly clockwise due to the distal curve of the structure. To lower rotation resistance, we recommend leaving the R-DLT free to rotate. Second, the more stringent definition of DLT malpositioning used here may be responsible for the higher malposition rates. Even in cases where malpositioning was noted, it was not always the case that left bronchial intubation using a R-DLT during OLV exhibited poor clinical performance. Retrospectively, most cases of R-DLT malpositioning were attributable to a position in which the carina is partly visible or to bronchial cuff herniation, which did not affect the mechanical ventilation. In addition, the malpositioning could be corrected easily and rapidly via bronchoscopy. Unlike other studies, we did not apply suction to R-DLTs [12].

A secondary outcome of our study was safety, which was determined according to damage to the airway. Risk factors for vocal cord and bronchial injury include physical contact with the tube and the cuff [12]. In our study, the numbers of patients with bronchial injuries or vocal cord injuries were comparable in both groups, and most airway injuries were minor. For example, tracheal redness could be observed at the site where the angled tip of the R-DLT touched the wall during bronchial insertion. When the lateral wall pressure of the bronchial cuffs is greater than the mean capillary perfusion pressure, bronchial mucosa injury may occur in a few hours [12]. In our study, to lower the pressure of the bronchial cuffs, we used a stethoscope to listen to the trachea for air leaks and injected the smallest amount of air necessary to achieve an adequate tracheal seal; we also monitored cuff pressure.

The incidences of postoperative sore throat and hoarseness were not significantly different between the groups receiving right- or left-side bronchial intubation with a R-DLT, mainly because we left the R-DLT free to rotate to reduce the pressure in the throat. In addition, when a R-DLT is inserted into a patient’s airway, body heat may decrease the stiffness of the DLT. In a previous study, warming the distal part of the DLT appeared to be effective for reducing some airway complications [16, 17] . A previous study reported that 3% of the patients were hoarse for more than 5 postoperative days [18]. In our study, no patient had hoarseness on the 4th day after the operation, although the limited size of our study might explanation the lack of patients with long-lasting hoarseness.

This study has certain limitations. First, we cannot completely exclude the possibility that sore throat might be caused by the laryngeal mask and the flexible bronchoscope itself. In our study, experienced pulmonologists and anesthesiologists performed all of the procedures to minimize airway injury. Second, numerous patients refused to undergo postoperative flexible bronchoscope examination. Thus, vocal cord injuries and bronchial injuries were assessed only immediately after extubation. Third, in our study, the sample size was too small to detect rare cases of severe airway damage (e.g., serious tracheal wall rupture).

## Conclusions

In conclusion, repositioning R-DLTs from the right main bronchus into the left main bronchus showed good clinical performance without causing additional injury with patients in the lateral position during surgery. Thus, this approach may offer a solution to right upper lobe occlusion by a R-DLT during surgery.

## Additional files

Additional file 1: **Table S1**. Factors related to the initial tracheal intubation in the dorsal decubitus position. (DOCX 18 kb)

Additional file 2: **Table S2**. Pulmonary mechanics during ventilation. (DOC 37 kb)

Additional file 3: **Table S3**. Arterial blood gas analysis. (DOC 38 kb)

## Abbreviations

FOB: fiberoptic bronchoscope
L-DLT: left -sided double-lumen tube
OLV: one-lung ventilation
R-DLT: right-sided double-lumen tube
