# Supplementary material for: A method for addressing right upper lobe obstruction with right-sided double-lumen endobronchial tubes during surgery: a randomized controlled trial
Source: BMC Anesthesiol. 2018 Sep 18;18:130. doi: 10.1186/s12871-018-0596-3 (PMC6142378; doi:10.1186/s12871-018-0596-3)
Supplement: Supplementary file 1 — Table S1. Factors related to the initial tracheal intubation in the dorsal decubitus position. (DOCX 18 kb) [file 12871_2018_596_MOESM1_ESM.docx]

|  | **Group R (n=30)** | **Group L (n=30)** | **P Value** |
| --- | --- | --- | --- |
| Time for tracheal intubation and R-DLT positioning(s) | 50.0(46.5-62.7) | 57.0(48.0-74.8) | 0.206 ^a^ |
| Number of laryngoscopies (1/2) | 29(96.7)/1(3.3) | 28(93.3)/2(6.7) | 1.000 ^b^ |
| Correct position after intubation (Optimal/Sub-optimal/Misplaced/Unsatisfactory) | 20(67.7)/7(23.3)/2 (6.7)/1(3.3) | 22(73.3)/5(16.7)/1 (3.3)/2(6.7) | 0.787 ^b^ |
| Number of tracheal intubation attempts (1/2/3) | 21(70.0)/7(23.3)/  2(6.7) | 24(80.0)/5(16.7)/1 (3.3) | 0.642 ^b^ |

**Table S1 Factors related to the initial tracheal intubation in the dorsal decubitus position**

^a^ Median [IQR] (range) of the non-normal variables (Kolmogorov–Smirnov test, P<0.05) and the Mann–Whitney rank sum test).

^b^ Numbers (%) for the categorical variables, Fisher’s exact test.
