# Supplementary material for: A method for addressing right upper lobe obstruction with right-sided double-lumen endobronchial tubes during surgery: a randomized controlled trial
Source: BMC Anesthesiol. 2018 Sep 18;18:130. doi: 10.1186/s12871-018-0596-3 (PMC6142378; doi:10.1186/s12871-018-0596-3)
Supplement: Supplementary file 2 — Table S2. Pulmonary mechanics during ventilation. (DOC 37 kb) [file 12871_2018_596_MOESM2_ESM.doc]

| **Table S2 Pulmonary mechanics during ventilation** | | | |
| --- | --- | --- | --- |
|  | **Group R (n=30)** | **Group L (n=30)** | **P Value** |
| Peak pressure (cm H2O) |  |  |  |
| T_1_ | 16.7(4.6) | 18.7(4.8) | 0.107 ^a^ |
| T_2_ | 22.7(4.4) | 23.4(4.0) | 0.527 ^a^ |
| T_3_ | 22.8(4.1) | 23.4(3.9) | 0.544 ^a^ |
| T_4_ | 22.8(4.0) | 23.4(3.9) | 0.540 ^a^ |
| Plateau pressure (cm H2O) |  |  |  |
| T_1_ | 13.7(3.7) | 15.3(4.4) | 0.122 ^a^ |
| T_2_ | 18.7(3.4) | 19.5(3.6) | 0.379 ^a^ |
| T_3_ | 18.7(3.3) | 19.4(3.6) | 0.415 ^a^ |
| T_4_ | 18.7(3.4) | 19.5(3.6) | 0.419 ^a^ |
| Static compliance (ml/cm H2O) | |  |  |
| T_1_ | 38.1(9.6) | 33.8(13.0) | 0.153 ^a^ |
| T_2_ | 25.0(6.9) | 23.4(7.2) | 0.383 ^a^ |
| T_3_ | 25.1(6.5) | 23.4(7.2) | 0.330 ^a^ |
| T_4_ | 25.1(6.6) | 23.5(6.8) | 0.370 ^a^ |

^a^ Mean (SD) of the normal variables, independent Student’s t-tests.

T_1_, before OLV; T_2_, 5 min after OLV; T_3_, 15 min after OLV; T_4_, 30 min after OLV.
