# Supplementary material for: A method for addressing right upper lobe obstruction with right-sided double-lumen endobronchial tubes during surgery: a randomized controlled trial
Source: BMC Anesthesiol. 2018 Sep 18;18:130. doi: 10.1186/s12871-018-0596-3 (PMC6142378; doi:10.1186/s12871-018-0596-3)
Supplement: Supplementary file 3 — Table S3. Arterial blood gas analysis. (DOC 38 kb) [file 12871_2018_596_MOESM3_ESM.doc]

^a^ Mean (SD) of the normal variables, independent Student’s t-tests.

**Blood gas analysis was performed before and 10 min after lung collapse.**

| **Table S3 Arterial blood gas analysis** | | | |
| --- | --- | --- | --- |
|  | **Group R (n=30)** | **Group L (n=30)** | **P Value** |
| pH before OLV | 7.35(0.04) | 7.35(0.05) | 0.593 ^a^ |
| Paco_2_ before OLV (mmHg) | 39.8(5.2) | 41.8(6.1) | 0.174 ^a^ |
| Pao_2_ before OLV (mmHg) | 450.6(26.8) | 438.1(32.2) | 0.106 ^a^ |
| Saturation during OLV (%) | 99.7(0.50) | 99.4(0.45) | 0.072 ^a^ |
| pH during OLV | 7.34(0.04) | 7.32(0.39) | 0.398 ^a^ |
| Paco_2_ during OLV (mmHg) | 42.3(4.1) | 42.6(4.4) | 0.783 ^a^ |
| Pao_2_ during OLV (mmHg) | 179.9(22.7) | 177.7(35.0) | 0.772 ^a^ |
| Saturation during OLV (%) | 97.8(2.2) | 97.7(2.2) | 0.917 ^a^ |
